# Who should operate patients presenting with emergent colon cancer? A comparison of short- and long-term outcome depending on surgical sub-specialization

**DOI:** 10.1186/s13017-023-00474-y

**Published:** 2023-01-09

**Authors:** Örvar Arnarson, Ingvar Syk, Salma Tunå Butt

**Affiliations:** Department of Surgery, Skane University Hospital Malmo, Lund University, Lund, Sweden

**Keywords:** Emergency surgery, Colorectal cancer, Sub-specialization, Large bowel obstruction

## Abstract

**Background:**

Colorectal cancer presents as emergencies in 20% of the cases. Emergency resection is associated with high postoperative morbidity and mortality. The specialization of the operating team in the emergency settings differs from the elective setting, which may have an impact on outcome. The aim of this study was to evaluate short- and long-term outcomes following emergent colon cancer surgery depending on sub-specialization of the operating team.

**Methods:**

This is a retrospective population study based on data from the Swedish Colorectal Cancer Registry (SCRCR). In total, 656 patients undergoing emergent surgery for colon cancer between 2011 and 2016 were included. The cohort was divided in groups according to specialization of the operating team: (1) colorectal team (CRT); (2) emergency surgical team (EST); (3) general surgical team (GST). The impact of specialization on short- and long-term outcomes was analyzed.

**Results:**

No statistically significant difference in 5-year overall survival (CRT 48.3%; EST 45.7%; GST 42.5%; *p* = 0.60) or 3-year recurrence-free survival (CRT 80.7%; EST 84.1%; GST 77.7%21.1%; *p* = 0.44) was noted between the groups. Neither was any significant difference in 30-day mortality (4.4%; 8.1%; 5.5%, *p* = 0.20), 90-day mortality (8.8; 11.9; 7.9%, *p* = 0.37) or postoperative complication rate (35.5%, 35.9 30.7, *p* = 0.52) noted between the groups. Multivariate analysis adjusted for case-mix showed no difference in hazard ratios for long-term survival or postoperative complications. The rate of permanent stoma after 3 years was higher in the EST group compared to the CRT and GST groups (34.5% vs. 24.3% and 23.9%, respectively; *p* < 0.0.5).

**Conclusion:**

Surgical sub-specialization did not significantly affect postoperative complication rate, nor short- or long-term survival after emergent operation for colon cancer. Patients operated by emergency surgical teams were more likely to have a permanent stoma after 3 years.

**Supplementary Information:**

The online version contains supplementary material available at 10.1186/s13017-023-00474-y.

## Introduction

Colorectal cancer (CRC) is among the most common cancers globally and counting for over 7000 new cases in Sweden annually [1]. Apart from tumor biology and stage, survival is dependent on various factors including the quality of the surgical resection, number of examined lymph nodes and adequate adjuvant therapy [2–6]. Approximately 20% of the colon cancer cases present as emergencies, in which acute resection is associated with increased perioperative morbidity and mortality, but also impaired long-term survival compared to elective cases, independent of tumor stage [7, 8]. Patients undergoing emergency surgery tend to have more advanced cancer with higher T-stage and N-stage compared to electively operated patients [9]. Radical resection rate has also been shown to be lower among emergency presented cases [10].

In Sweden, as in many western countries, surgical care is characterized by centralization and sub-specialization. Most high-volume hospitals have both colorectal teams with colorectal surgeons managing all elective cases of CRC and emergency surgery teams dealing with a wide spectrum of emergency cases, including emergent colon cancer surgery. Smaller- or low-volume hospitals usually do not have this division of specialized teams, and colorectal surgery as well as emergency surgery is performed by general surgeons with or without colorectal specialization. Some studies suggest that surgical specialization, hospital volume and caseload are important prognostic factors in elective colorectal cancer surgery [11–14], whereas the impact of surgical specialization on emergent colon cancer is more elusive [15–18].

The aim of this study was to evaluate the impact of surgical specialization on short- and long-term outcomes in patients undergoing emergent colon cancer surgery. Primary endpoints were 5-year overall survival and 3-year recurrence-free survival. Secondary endpoints were rate of radical resections, postoperative complication rate, 30- and 90-day postoperative mortality and stoma rate at 3 years.

## Methods

This was a retrospective study, based on the Swedish Colorectal Cancer Registry (SCRCR), in which all primary tumors of invasive adenocarcinomas are registered. Hence, no recurrent cancer was included in this study. In the south region of Sweden, with approximately 1.8 million inhabitants, an addition has been made in the registry, regarding whether the patient was operated on by a colorectal team, an emergency surgical team or not applicable. The latter refers to smaller hospitals lacking specialized surgical teams but handled by general surgeons, often with a broad surgical experience. In total, 8 hospitals in the region performed surgery for colorectal cancer during the study period, of which 5 had sub-specialized teams (colorectal and emergency teams) and 3 did not. All patients operated on with emergent resection for colon cancer in the south region of Sweden between 2011 and 2016 were identified via SCRCR and were included in the study. Whether the surgeon was a qualified specialist in surgery, sub-specialist in colorectal surgery or general surgery was noted. In the SCRCR, an emergency resection was defined as a resection performed in a patient admitted via the emergency department due to acute symptoms emanating from the tumor and requiring immediate resection. No data on duration of symptoms were available in the register. The coverage in SCRCR, as compared to the Swedish cancer registry where registration is compulsory, was 98.5–99.6% during the study period. The cohort was divided into three categories: (1) operated by colorectal team (CRT); (2) operated by emergency surgical team (EST); and (3) operated by general surgical team (GST). Patients that did not undergo primary resection of the tumor, but were operated with intestinal by-pass, bowel deviation, endoluminal stent or just open and close procedure were excluded from the study. Cases operated in the specialized hospitals with missing data regarding sub-specialization of operating team were excluded, whereas missing data on specialization in the small hospitals were considered operated by general surgical teams.

Date of death was registered by linking to the Swedish Population Registry, which is continuously updated. Last retrieval from both registers was performed 3^rd^ mars 2020, giving a mean follow-up time of 4.2 (S.D. ± 1.7) years. Data retrieved from the SCRCR included: patient demographics, tumor characteristics, operative details, complete pathology results, detailed information on postoperative events, and date and localization of any recurrence. All patients were routinely followed up with a CT scan at 3 years, whereas the number of examinations up to three years differed among centers. Further, if the patients were operated on with a diverting ileostomy or a permanent stoma and whether they had undergone reversal of any diverting ileostomy within 3 years postoperatively were noted. Postoperative complications were graded by the Clavien–Dindo classification system, and grade 3b or higher was defined as severe complications. Patients were considered radical resected if judged both macroscopically radical by the surgeon and microscopically radical according to the pathology report. Doubtful and undefinable resections were considered as not radical. Curative operation was defined as radical resection in M0 patient. Whether the operation started after daytime hours (i.e., 16:00) on any day of the week was noted.

### Statistical analysis

Categorical variables are reported as numbers and proportion; continuous variables are reported as median and range. Comparisons of continuous and categorical variables were analyzed with Kruskal–Wallis and Chi^2^ test, respectively. *p* values less than 0.05 were considered statistically significant, and 95% confidence intervals (CI) are presented when appropriate.

Kaplan–Meier curves were used to describe overall (OS) and recurrence-free survival (RFS) rates, which were analyzed with log rank test to determine statistical significance. OS and RFS were defined as time from date of operation to death of any cause and date of recurrence, respectively. Patients alive and without recurrence were censored at last follow-up. Association of well-known confounders on postoperative mortality and long-term survival was examined with univariate analysis. Variables with *p* value of 0.1 or less were entered in a multivariate model to determine predictors for those outcomes. Regarding postoperative mortality, logistic regression analysis was used, whereas for overall and recurrence-free survival Cox proportional hazards regression analysis was used.

Statistical analyses were performed using SPSS (IBM SPSS version 25, Armonk, NY, USA). Survival curves were generated using Stata (release 17; Stata Statistical Software, College Station, TX, USA).

## Results

A total of 699 patients were identified as having undergone emergent colonic resection between 2011 and 2016 due to cancer with acute symptoms. Of these, 43 patients were excluded because of missing data. Hence, a cohort of 656 patients were included in the study of which 319 were operated by colorectal teams (CRT), 210 by emergency surgical teams (EST) and 127 by general surgical teams (GST). Median age was 75 (range 32–101), and 319 (48.6%) were male and 337 (51.4%) females.

Patient demography and tumor characteristics are presented in Table 1. There was no significant difference in gender or age between the three groups. Neither were there any significant differences in the proportion of high- grade or mucinous type nor T- or M-stage, whereas for N-stage, a borderline statistical difference was noted (*p* = 0.06). Notably, the rate of preoperative staging regarding liver and lung metastases was higher in the CRC group (90.3%), compared to the EST and GST groups (66.7% and 63.8%, respectively, *p* < 0.001), although proper M-staging was done in all but 4 patients during hospitalization. The proportion of patients with ASA score 3 and 4 was higher in the EST group (53.8%) compared with the CRT group (43.9%) and the GST group (40.9%) (*p* < 0.05) (Table 1).

**Table 1:** Patient and tumor characteristics stratified on specialization of operating team

	CRT* (N = 319)		EST** (N = 210)		GST*** (N = 127)		*p* value^#^
	*n*	(%)	*n*	(%)	*N*	(%)	
*Gender*							
Male	147	(46.1)	111	(52.9)	61	(48.0)	0.31
Female	172	(53.9)	99	(47.1)	66	(52.0)	
*Age*							
< 66	77	(24.1)	52	(24.8)	27	(21.3)	0.75
66–80	149	(46.7)	91	(43.3)	60	(47.2)	0.70
> 80	93	(29.2)	67	(31.9)	40	(31.5)	0.77
*ASA score*							
ASA 1–2	175	(54.9)	80	(38.1)	74	(58.3)	< 0.05
ASA 3	126	(39.5)	92	(43.8)	40	(31.5)	< 0.05
ASA 4	14	(4.4)	21	(10.0)	12	(9.4)	< 0.05
Missing	4	(1.3)	17	(8.1)	1	(0.8)	–
*Indication for surgery*							
Obstruction	261	(80.8)	155	(73.8)	97	(76.4)	0.08
Bleeding	13	(4.1)	9	(4.3)	4	(3.1)	0.87
Perforation	36	(11.3)	29	(13.8)	19	(15.0)	0.50
Other	9	(2.8)	17	(8.1)	7	(5.5)	< 0.05
*Tumor location*							
Appendix	4	(1.3)	4	(1.9)	3	(2.4)	0.68
Right colon	115	(35.1)	84	(40.5)	55	(43.3)	0.31
Transverse colon	34	(10.7)	17	(8.1)	16	(12.6)	0.39
Left colon	166	(52.0)	104	(49.5)	53	(41.7)	0.14
*pT stage*							
T1–T2	13	(4.1)	6	(2.9)	7	(5.5)	0.48
T3	137	(42.9)	89	(42.4)	56	(44.1)	0.95
T4	167	(52.4)	114	(54.3)	64	(50.4)	0.78
TX	2	(0.6)	1	(0.5)	0	(0.0)	–
*pN stage*							
N0	106	(33.2)	72	(34.3)	56	(44.1)	0.06
N1–2	211	(66.1)	136	(64.8)	68	(53.5)	< 0.05
Missing	2	(0.6)	2	(1.0)	3	(2.4)	–
*M-stage*^¤^							
M0	246	(77.1)	164	(78.1)	97	(76.4)	0.93
M1	73	(22.9)	45	(21.4)	27	(21.3)	0.09
Missing	0	(0.0	1	(0.5)	3	(2.4)	–
*Surgical specialization*							
CR^§^	278	(87.7)	60	(29.4)	71	(56.3)	< 0.05
Non-CR	39	(12.3)	144	(70.6)	55	(43.7)	< 0.05

^*^CRT Colorectal team

^**^EST Emergency surgical team

^***^GST General surgical team

^#^Chi-square test

^¤^Based on pre- or postoperative CT scanning

^§^*CR* Colorectal

*﻿ASA score* American Society of Anaesthesiologists classification

Colonic obstruction was the most common indication for surgery, 80.8%, 73.8% and 76.4% in CRT, EST and GST groups, respectively (*p* = 0.08), Table 1. There was no difference in tumor location, but 11 patients in the CRC group underwent anterior resection compared to none of the patients in the other two groups. Otherwise, there was no significant difference in type of resection, data not shown. Laparoscopic approach was used in only 11 cases without any significant difference between the groups. There was no significant difference between the three groups whether the patients were operated during night hours or not. Formal qualification differed among the groups, but all resections were performed by specialists in surgery and thus having at least 5 years of surgical experience (Table 1).

### Primary endpoints

**Fig. 1:**
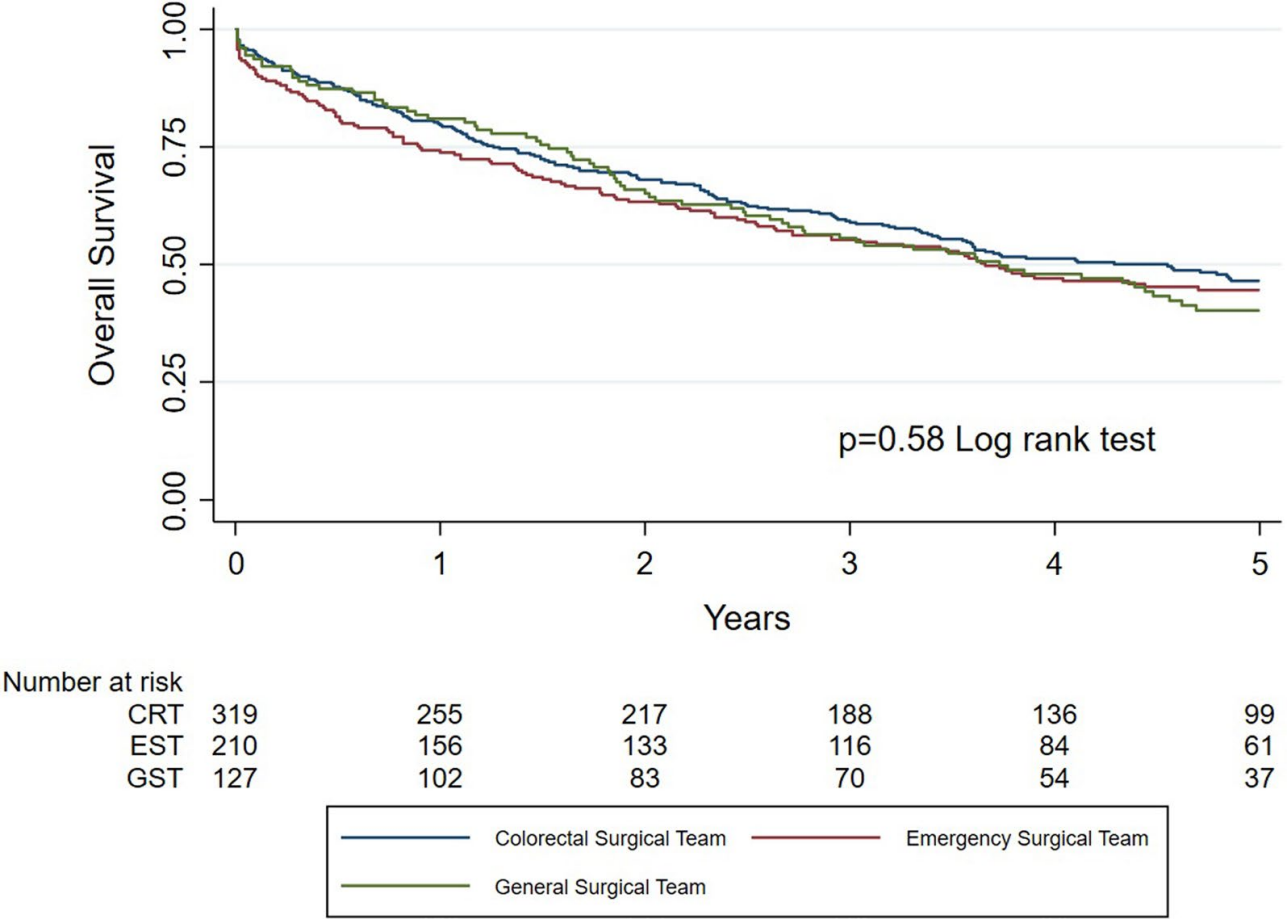
Kaplan–Meier survival estimates for overall survival stratified by specialization of the surgical team

**Fig. 2:**
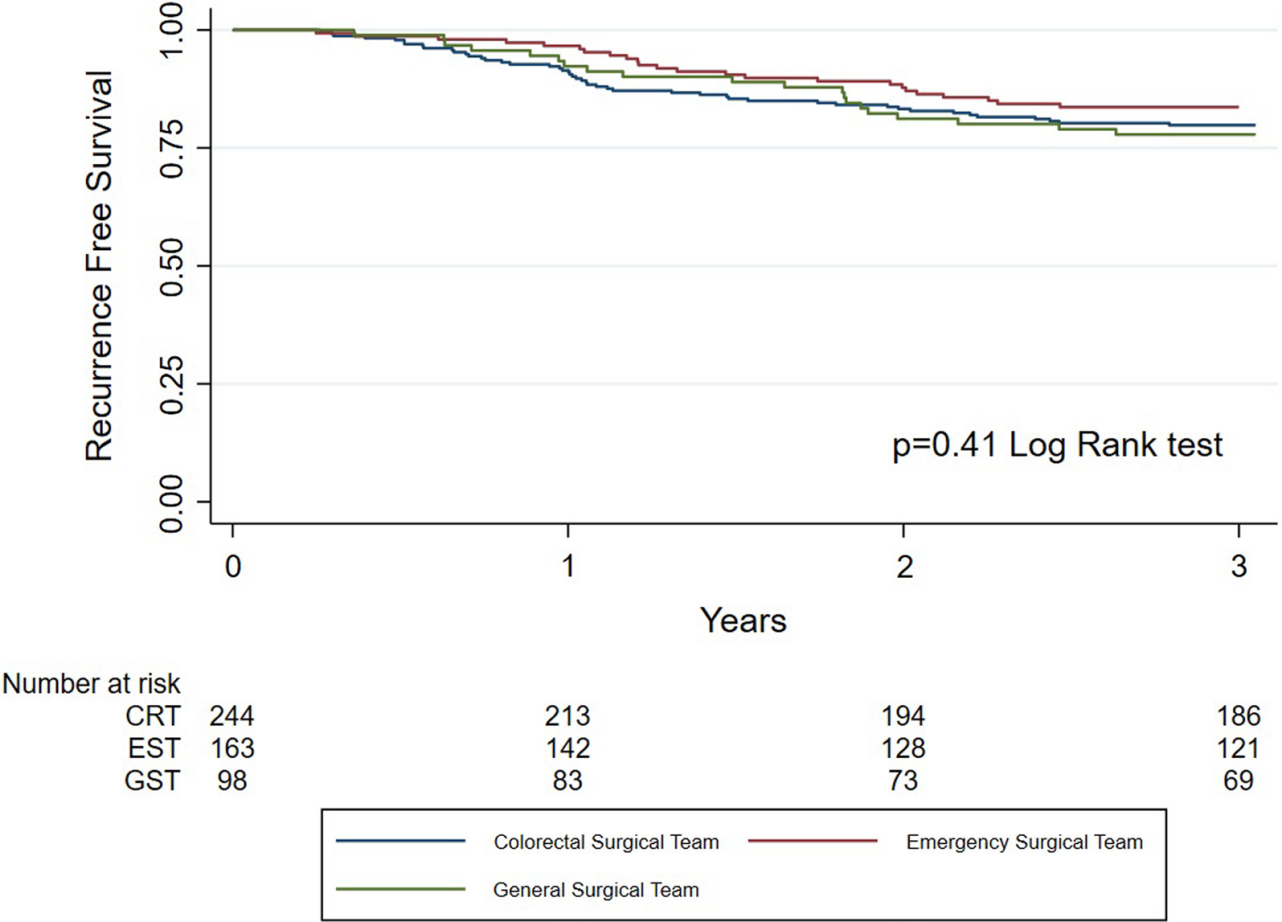
Kaplan–Meier survival estimates for recurrence-free survival stratified by specialization of the surgical team. M0 patients only

Five-year overall survival rates did not differ statistically significant depending on operating team and was 48.3% in the CRT group, 45.7% in the EST group and 42.5% in the GST group (*p* = 0.57) (Table 2 and Fig. 1). Three-year recurrence-free survival in M0 patients did not differ either and was 80.1% in the CRT group, 84.1% in the EST group and 77.3% in the GST group (*p* = 0.44) (Table 2 and Fig. 2).

**Table 2:** 5-year overall mortality and 3-year recurrence-free survival following emergent resection of colon cancer, stratified on specialization of operating team

	CRT (%)	EST (%)	GST (%)	*p* value^#^
5-year OS	48.3	45.7	42.5	0.57
3-year RFS*	80.1	84.1	77.3	0.44

*CRT* Colorectal team, *EST* Emergency surgical team, *GST* General surgical team

^*^M0 at diagnosis only

^#^Log rank

*OS* Overall survival, *RFS* Recurrence-free survival

Multivariate COX proportional risk analysis showed no difference in impact of surgical specialization on 5-year OS or 3-year RFS (Table 3). Selection of variables adjusted for, according to principles given in methods, is presented in Additional file 1: Table S1.

**Table 3:** Multivariate COX proportional hazard model

	5-year mortality			Recurrence within 3 years		
	HR	95% CI	*p* value	HR	95% CI	*p* value
*Age*						
< 65	1.0			1.0		
65–74	1.2	(0.8–1.6)	0.4	1.2	(0.7–2.1)	0.52
75–84	1.8	(1.3–2.5)	0.00	1.0	(0.6–1.8)	0.95
> 85	3.6	(2.5–5.1)	0.00	0.5	(0.2–1.3)	0.15
*ASA score*						
1–2	1.0			1.0		0.90
3	1.8	(1.4–2.3)	0.00	1.0	(0.6–1.5)	0.50
4	4.0	(2.8–5.9)	0.00	1.4	(0.5–3.7)	0.60
*T-stage*						
1–2	1.0			1.0		
3	1.4	(0.7–3.0)	0.34	1.1	(0.3–4.6)	0.90
4	2.4	(1.1–4.9)	0.2	1.4	(0.3–5.9)	0.70
*N-stage*						
0	1.0			1.0		
1–2	1.6	(1.2–2.1)	< 0.05	2.9	(1.7–4.9)	< 0.05
*M-stage*						
0	1.0					
1	2.9	(2.3–3.7)	0.00			
*Surgical specialization*						
CRT	1.0			1.0		
EST	1.0	(0.7–1.3)	0.88	0.7	(0.4–1.1)	0.15
GST	1.2	(0.9–1.6)	0.24	1.3	(0.8–2.2)	0.36

Hazard ratios (HR) for 5-year overall mortality and recurrence within 3 years following emergent resection of colon cancer

*CST* colorectal team, *EST* emergency surgical team, *GST* general surgical team, *ASA score* American Society of Anaesthesiologists classification

### Secondary endpoints

One or more postoperative complications were registered in 232 (35.4%) patients. Neither the total complication rate nor the rate of severe postoperative complications (CD ≥ 3b) differed between the groups, and there was no difference in thirty- or ninety-day mortality. Neither was there any difference in whether the patients received adjuvant or palliative treatment (Table 4).

**Table 4:** Perioperative outcome and oncological treatment, stratified on specialization of surgical team

	CRT *N* = 319		EST *N* = 210		GST *N* = 127		*p* value^#^
	*n*	(%)	*n*	(%)	*N*	(%)	
*Postoperative complications*							
All	113	(35.5)	75	(35.9)	39	(30.7)	0.53
Severe (≥ CD* 3b)	55	(17.2)	46	(21.9)	22	(17.3)	0.37
*Postoperative mortality*							
30-day mortality	14	(4.4)	17	(8.1)	7	(5.5)	0.20
90-day mortality	28	(8.8)	25	(11.9)	10	(7.9)	0.37
*Stoma*							
At primary operation	148	(46.4)	88	(41.9)	43	(33.9)	< 0.05
Diverting ileostomy	54	(16.9)	12	(5.7)	11	(8.7)	< 0.05
Sigmoidostomy	33	(10.3)	30	(14.3)	13	(10.2)	0.13
Other	61	(19.1)	46	(21.9)	19	(15.0)	0.25
Stoma after 3 years**	46/189	(24.3)	40/116	(34.5)	17/71	(23.9)	< 0.05
*Chemotherapy*							
Adjuvant	133	(41.7)	77	(36.7)	51	(40.2)	0.51
Palliative	28	(8.8)	21	(10.0)	11	(8.7)	0.87
Operated outside office hours***	84	(26.3)	59	(28.1)	34	(26.8)	0.75

*CST* colorectal team, *EST* emergency surgical team, *GST* general surgical team

^#^Chi-square test

^*^CD Clavien-Dindo classification of postoperative complication

^**^Permanent stomas in patients alive after 3 years

^***^Operation performed between 16 and 08 o’clock

Surgical specialization was not predictive for 30-day mortality (OR = 1.4 [95% CI 0.6–3.2] and 1.2 [95% CI 0.4–3.2]) or 90-day mortality (OR = 1.0 [95% CI 0.3–2.0] and 0.8 [95% CI 0.3–1.8]) in multivariate logistic regression analyses. Only high ASA score was predictive for 30-day mortality, whereas besides high ASA score, both high age and metastasized disease were risk factors for 90-day mortality (Table 5). Selection of variables adjusted for, according to principles given in methods, is presented in Additional file 1: Table S2.

**Table 5:** Risk factors for 30- and 90-day postoperative mortality following emergent resection of colon cancer

	30-day mortality			90-day mortality		
	OR	(95% CI)	*p* value	OR	(95% CI)	*p* value
*Age*						
< 65	1.0			Ref		
65–74	0.4	(0.1–1.9)	0.29	0.7	(0.2–2.2)	0.53
75–84	1.6	(0.5–4.7)	0.40	1.9	(0.7–4.8)	0.21
> 85	1.9	(0.6–6.0)	0.26	4.2	(1.6–11.5)	< 0.05
*ASA score*						
1–2	1.0			Ref		
3	3.9	(1.4–10.9)	< 0.05	4.0	(1.7–9.1)	< 0.05
4	18.4	(5.7–58.7)	< 0.05	18.8	(7.0–53.5)	< 0.05
*M-stage*						
0				Ref		
1				2.9	(1.4–5.8)	< 0.05
*Indication for surgery*						
Obstruction	1.0					
Bleeding	0.8	(0.2–4.0)	0.80	1.8	(0.5–5.8)	0.35
Perforation	1.2	(0.4–3.0)	0.77	1.7	(0.7–3.6)	0.16
Other	1.1	(0.3–4.5)	0.86	2.1	(0.6–5.9)	0.22
*Surgical specialization*						
CRT	1.0			Ref		
EST	1.4	(0.6–3.1)	0.41	1.0	(0.5–2.0)	0.96
GST	1.1	(0.4–2.9)	0.89	0.8	(0.3–1.8)	0.60

Multivariate logistic regression

*CST* colorectal team, *EST* emergency surgical team, *GST* general surgical team, *ASA score* American Society of Anaesthesiologists classification

A total of 279 of the 656 patients (42.5%) were operated on with a stoma: 46.4% in the CRT group; 41.9% in the EST group; and 33.9% in the GST group (*p* < 0.05). The use of diverting ileostomy was more often used by colorectal teams, *i.e.,* in 16.9% (54/319) compared to 5.7% (12/210) in the EST group and 8.7% (11/127) in the GST group, (*p* < 0.05). In turn, patients in the CRT group had higher rate of stoma reversal and of all patients alive after 3 years, 24.3% (46/189) patients in the CRT group had a permanent stoma compared with 34.5% (40/116) in the EST group and 23.9% (17/71) in the GST group (*p* < 0.05) (Table 4).

The number of examined lymph nodes was significantly less in the GST group compared with the other groups. Moreover, the rate of microscopically radical resection of the primary tumor did not differ between groups (Table 6).

**Table 6:** Primary oncological and pathology results stratified on specialization of surgical team

	CRT		EST		GST		*p* value
	*N* = 319		*N* = 210		*N* = 127		
Radical resection (R0), *n* (%)	289	(90.9)	178	(85.2)	114	(89.8)	0.12^#^
Curative operation, *n* (%)	228	(71.5)	147	(70.0)	89	(70.1)	0.92^#^
Examined lymph nodes, Mean, (S.D.)	26.6	(13.9)	26.9	(17.7)	22.8	(11.9)	< 0.05^##^

*CST* colorectal team, *EST* emergency surgical team, *GST* general surgical team

^#^Chi-square test

^##^Kruskal–Wallis test

## Discussion

This study shows that neither risk of recurrence nor survival after emergent colon cancer resection was influenced by the specialization of the surgical team performing the operation. No difference in postoperative morbidity or mortality rate was noted. Patients operated by emergency teams had a higher rate of permanent stoma after 3 years compared with patients operated by colorectal surgical teams or general surgeons. This may reflect that colorectal surgeons are more prone to opt for primary anastomosis and diverting ileostomy in left-sided resections.

The rate of microscopically radical resections did not differ between the groups. In contrast, a difference in number of examined lymph nodes was noted, which, however, did not reflect in any difference in risk of recurrence. The difference was small and could as well depend on the pathology as the surgery. Further, all groups had totally sufficient numbers of examined lymph nodes and we perceive the noted difference not to be of clinical importance. Notably, a borderline difference in N-stage, with more N0 in the GST group, was noted. Although the reason for this is elusive, it might reflect a stage migration albeit the sufficient number of examined lymph nodes as any difference in N-stage depending on geography is less likely. Nevertheless, as the whole groups of patients were analyzed, any stage migration should not impact the primary endpoints, survival and recurrence rate.

Several studies have shown an association between outcome and surgical sub-specialization and surgical volume, respectively, in elective surgery for colon cancer [12, 19–22], although not all were consistent. For example, Hall et al. performed a retrospective registry-based study of 21,432 patients who had undergone elective operation for colon cancer and 5893 operated on for rectal cancer either by colorectal specialists or general surgeons. Colorectal surgeons performed 16.3% of the colon and 27% of the rectal resections. They found no difference in overall 5-year disease-specific survival (DSS) between the specialties except in stage II rectal cancer in a multivariate analysis. When the analysis was limited to high-volume surgeons only, the results remained the same [2].

The impact of specialization and caseload in the emergency settings is much less studied and unclear. Kwan et al. studied the impact of hospital volume on 30-day postoperative mortality following emergency colorectal surgery in 864 patients, of which 63.8% had colon cancer, operated in 15 different hospitals. The hospitals were grouped into low, medium and high operative volume according to caseload. The colorectal POSSUM scoring system was used to adjust for difference in case-mix in the study. Thirty-day mortality was 16.3% without any statistical difference in mortality between hospitals of different case volume [23], a finding in line with our result. Kulyat et al. studied short-term outcomes in patients undergoing emergent colectomies by colorectal surgeons compared to general or emergency care surgeons in 3 academic hospitals. A propensity score matching was performed with 238 patients in each group. Operations performed by colorectal surgeons were associated with significantly lower rates of 30-day mortality (6.7% vs 16.4%, *p* = 0.001) and postoperative morbidity (45.0% vs 56.7%, *p* = 0.009). However, only 13.0% of the patients had a malignant disease [24]. A large population-based registry study from the UK showed that emergency laparotomy performed by consultants without a special interest in colorectal surgery had an increased adjusted 30-day mortality risk (OR 1.23, 95 CI 1.13–1.33) as well as increased risk of re-operation (OR 1.13, CI 1.05–1.20) compared to consultants with special colorectal interest [25].

A Swedish registry-based retrospective study on 13,365 patients operated on for colon cancer between 2007 and 2010 focused on formal competence of the most senior surgeon attending the procedure irrespective of surgical team or hospital volume, of which 21.9% were emergency procedure. The result showed superior five-year overall survival in patients operated by colorectal surgeons (36.6%) compared to general surgeons (33.4%) (*p* < 0.05). However, after adjusting for 30- and 90-day mortality, no statistically significant difference was noted. Hence, the difference in long-term survival was explained by a lower postoperative mortality in the group of patients operated by colorectal surgeons. However, it is unclear to what degree the improved postoperative mortality rate was dependent on the specialization of the surgeon or the competence of the whole team, caring for the patient postoperatively. Probably this result was also affected by a case-mix, such as more frail and severely ill patients, *e.g.,* with peritonitis, had to be operated during on-call and thus by younger less qualified surgeons [26].

Moreover, high-volume hospitals not only have more colorectal specialist, but also better intensive care and a lower rate of failure to rescue (FTR) [27–29] which reflects the rate of mortality after major complications. Postoperative complications greatly affect short- and long-term survival after surgery for colon cancer and even more so in patients operated on acutely [30]. Preoperative comorbidities, such as congestive heart failure and chronic renal failure, have been associated with higher rates of FTR in emergency general surgery [31]. These patients may neither tolerate fluid shifts nor the resuscitation required to restore physiologic parameters postoperatively [31]. Hospital factors also influence FTR. Multidisciplinary approach is needed for identification of at-risk patients, prevention of avoidable complications, recognition of unavoidable complications and prompt intervention in attempt to prevent avoidable death [31, 32]. Henneman et al. evaluated the association between structural hospital characteristics (hospital volume, teaching status and intensive care facilities (ICU) and FTR after colorectal cancer surgery. Only higher levels of ICU facilities were associated with lower FTR rates (OR 0.72; 95% CI 0.65–0.88) in multivariate analysis [28]. Intensive care in Sweden is generally of high quality and quite standardized between hospitals which might explain why we did not find any difference in in-hospital mortality between the groups in the present study.

The Union of International Cancer Control (UICC) recommends the evaluation of a minimum of 12 LNs for appropriate staging of patients with pN + disease [33]. Some previous studies report insufficient examined lymph nodes in the emergent setting [34], perhaps due to technical difficulties and instable patients [35], with a subsequent risk of not having adjuvant chemotherapy, as the indication for adjuvant chemotherapy is determined foremost by node positivity. However, also other risk factors for recurrence, including emergent operation constitute indications for adjuvant treatment [36]. In the present study, no difference in the proportion of patients given adjuvant chemotherapy was noted, albeit a numerical difference in N0 stage, probably due to that emergent resection is an indication for adjuvant chemotherapy.

The weakness of the present study is the retrospective design, implying a risk of selection bias although known confounders, such as ASA score, age and TNM stage were adjusted for in the multivariate analysis. Although the five larger hospitals had dedicated teams for colorectal and emergency surgery, there was some overlapping of surgeon´s specialization, especially in the GST where over half of the surgeons had colorectal qualification.

## Conclusion

Postoperative morbidity and mortality as well as long-term survival following emergent resection for colon cancer did not significantly differ depending on surgical specialization. Long-term survival and postoperative outcomes proved good in comparison to results reported in the literature, despite the differences in formal training and small volumes in some of the hospitals. However, permanent stoma rate was higher in patients operated by emergency surgeons. Further studies comprising more detailed data on comorbidity and management of complications and the impact on survival would be of value.

## Supplementary Information

**Additional file 1. Table S1.** Association between selected confounders and 5-year mortality and recurrence within 3 years. Univariate analysis. **Table S2.** Association between selected preoperative variables and postoperative mortality. Univariate analysis.

## Data Availability

The ethical approval for this study prohibits us from publishing any patient data on individual level.

## Abbreviations

SCRCR: Swedish Colorectal Cancer Registry
CRT: Colorectal team
EST: Emergency surgical team
GST: General surgeons
OS: Overall survival
RFS: Recurrence-free survival
ASA: American Society of Anaesthesiologists classification
CD: Clavien–Dindo
FTR: Failure to rescue
ICU: Intensive care unit
