# Supplementary material for: Who should operate patients presenting with emergent colon cancer? A comparison of short- and long-term outcome depending on surgical sub-specialization
Source: World J Emerg Surg. 2023 Jan 9;18:3. doi: 10.1186/s13017-023-00474-y (PMC9830814; doi:10.1186/s13017-023-00474-y)
Supplement: Supplementary file 1 — Additional file 1. Table S1. Association between selected confounders and 5-year mortality and recurrence within 3 years. Univariate analysis. Table S2. Association between selected preoperative variables and postoperative mortality. Univariate analysis. [file 13017_2023_474_MOESM1_ESM.docx]

Supplementary table 1. Association between selected confounders and 5-year mortality and recurrence within 3 years. Univariate analysis

|  | **5-year overall survival** | | |  | **3-year recurrence free survival**** | | |
| --- | --- | --- | --- | --- | --- | --- | --- |
|  | No. of patients | n (%) | *p* value* |  | No. Of patients | n (%) | *p* value* |
| Age  <64  65–74  75–84  >85 | 156  177  215  108 | 64 (18.2)  78 (22.2)  125 (35.5)  85 (24.1) | <0.05  <0.05  0.10  <0.05 |  | 114  131  172  90 | 24 (24.7)  31 (32.0)  34 (35.1)  8 (8.2) | .55  .13  .79  .01 |
| ASA score  1-2  3  4  Missing | 329  258  47  42 | 128 (36.4)  166 (47.2)  42 (11.9)  16 (4.5) | <0.05  <0.05  <0.05  .07 |  | 270  186  36  15 | 56 (57.7)  31 (32.0)  5 (5.2)  5 (5.2) | .33  .28  .40  0.16 |
| T-stage  1–2  3  4  Missing | 26  282  345  3 | 9 (2.6)  123 (34.9)  218 (61.9)  2 (0.6) | <0.05  <0.05  <0.05 |  | 23  245  238  1 | 2 (2.1)  41 (42.3)  54 (55.7)  0 (0.0) | .19  .18  .06 |
| N-stage  0  1-2  Missing | 234  415  7 | 95 (27.4)  252 (71.6)  5 (1.4) | <0.05  <0.05 |  | 218  284  5 | 21 (21.6)  75 (77.3)  1 (1.0) | .00  .00 |
| M-stage  0  1  Missing | 507  145  4 | 227 (64.5)  124 (35.2)  1 (0.3) | <0.05  <0.05 |  |  |  |  |
| Indication for surgery  Obstruction  Bleeding  Perforation  Other | 513  26  83  34 | 272 (77.3)  13 (3.7)  47 (13.4)  20 (5.7) | .54  .70  .56  .54 |  | 392  22  64  29 | 82 (84.5)  4 (4.1)  7 (7.2)  4 (4.1) | .06  .9  .08  .45 |
| Surgical specialisation  CRT  EST  GST | 319  210  127 | 165 (46.9)  114 (32.4)  73 (20.7) | .33  .83  .34 |  | 246  164  97 | 49 (50.5)  26 (26.8)  22 (22.7) | .66  .19  .32 |

*Pearson Chi square, **M0 patients only

Supplementary Table 2 Association between selected preoperative variables and postoperative mortality. Univariate analysis

|  | **Thirty-day mortality** | | | **Ninety-day mortality** | | |
| --- | --- | --- | --- | --- | --- | --- |
|  | No. of patients | n (%) | *p* value* | No. of patients | n (%) | *p* value* |
| Age  <65  65-74  75-84  >85 | 156  177  215  108 | 5 (13.2)  3 (7.9)  16 (42.1)  14 (36.8) | .11  <0.05  0.2  <0.05 | 156  177  215  108 | 7 (11.1)  7 (11.1)  23 (36.5)  26 (41.3) | .13  <0.05  .10  <0.05 |
| ASA score  1-2  3  4  Missing | 329  258  47  22 | 5 (13.2)  18 (47.4)  14 (36.8)  1 (2.6) | <0.05  0.30  <0.05  <0.05 | 329  258  47  22 | 8 (12.7)  33 (52.4)  21 (33.3)  1 (1.6) | <0.05  <0.05  <0.05  <0.05 |
| T-stage  1-2  3  4  Missing | 26  282  345  3 | 2(5.3)  15 (39.5)  21 (55.3)  0 | 0.67  .65  .73 | 26  282  345  3 | 2 (3.2)  23 (36.5)  38 (60.3)  0 (0.00) | .46  .74  .28  .20 |
| N-stage  N0  N1-2  Missing | 234  415  7 | 12 (31.6)  26 (68.4)  0 () | .59  .50 | 234  415  7 | 17 (27.0)  46 (73.0)  0 | =.15  .13  .09 |
| M-stage  0  1  Missing | 507  145  4 | 26 (68.4)  12 (31.6)  0 | .18  .15 | 507  145  4 | 38 (60.3)  25 (39.7)  0 | <0.05  <0.05  <0.05 |
| Indication for surgery  Obstruction  Bleeding  Perforation  Other | 513  26  83  34 | 25 (65.8)  2 (5.3)  7 (18.4)  4 (10.5) | 0.06  .67  .27  .13 | 513  26  83  34 | 40 (63.5)  4 (6.3)  13 (20.6)  6 (9.5) | <0.05  .31  <0.05  .10 |
| Surgical specialisation  CRT  EST  GST | 319  210  127 | 14 (36.8)  17 (44.7)  7 (18.4) | .13  .08  .88 | 319  310  127 | 28 (44.4)  25 (39.7)  10 (15.9) | .49  .17  .46 |
